# Familiality of Co-existing ADHD and Tic Disorders: Evidence from a Large Sibling Study

**DOI:** 10.3389/fpsyg.2016.01060

**Published:** 2016-07-19

**Authors:** Veit Roessner, Tobias Banaschewski, Andreas Becker, Judith Buse, Sina Wanderer, Jan K. Buitelaar, Joseph A. Sergeant, Edmund J. Sonuga-Barke, Michael Gill, Iris Manor, Ana Miranda, Fernando Mulas, Robert D. Oades, Herbert Roeyers, Hans-Christoph Steinhausen, Steven V. Faraone, Philip Asherson, Aribert Rothenberger

**Affiliations:** ^1^Department of Child and Adolescent Psychiatry and Psychotherapy, Dresden University of TechnologyDresden, Germany; ^2^Department of Child and Adolescent Psychiatry and Psychotherapy, Central Institute of Mental HealthMannheim, Germany; ^3^Department of Child and Adolescent Psychiatry and Psychotherapy, University Medical Center GöttingenGoettingen, Germany; ^4^Department of Cognitive Neuroscience, Radboud University Nijmegen Medical CenterNijmegen, Netherlands; ^5^Department of Clinical Neuropsychology, Vrije Universiteit AmsterdamAmsterdam, Netherlands; ^6^Developmental Brain Behaviour Laboratory, School of Psychology, University of SouthamptonSouthampton, UK; ^7^Department of Psychiatry, Trinity Centre for Health SciencesDublin, Ireland; ^8^ADHD Unit, Geha Mental Health CentrePetach-Tiqva, Israel; ^9^Department of Developmental and Educational Psychology, University of ValenciaValencia, Spain; ^10^Neuropediatrics Unit, La Fe University HospitalValencia, Spain; ^11^Department of Child and Adolescent Psychiatry, University of Duisburg-EssenEssen, Germany; ^12^Department of Clinical and Experimental Psychology, Ghent UniversityGhent, Belgium; ^13^Department of Child and Adolescent Psychiatry, University of ZurichZurich, Switzerland; ^14^Departments of Psychiatry, Neuroscience and Physiology, State University of New York Upstate Medical UniversityNew York, NY, USA; ^15^Institute of Psychiatry, King's College LondonLondon, UK

**Keywords:** ADHD, tic disorders, comorbidity, familiality, IMAGE, SDQ, CRS-L

## Abstract

**Background:** The association of attention-deficit/hyperactivity disorder (ADHD) and tic disorder (TD) is frequent and clinically important. Very few and inconclusive attempts have been made to clarify if and how the combination of ADHD+TD runs in families.

**Aim:** To determine the first time in a large-scale ADHD sample whether ADHD+TD increases the risk of ADHD+TD in siblings and, also the first time, if this is independent of their psychopathological vulnerability in general.

**Methods:** The study is based on the International Multicenter ADHD Genetics (IMAGE) study. The present sub-sample of 2815 individuals included ADHD-index patients with co-existing TD (ADHD+TD, *n* = 262) and without TD (ADHD–TD, *n* = 947) as well as their 1606 full siblings (*n* = 358 of the ADHD+TD index patients and *n* = 1248 of the ADHD-TD index patients). We assessed psychopathological symptoms in index patients and siblings by using the Strength and Difficulties Questionnaire (SDQ) and the parent and teacher Conners' long version Rating Scales (CRS). For disorder classification the Parental Account of Childhood Symptoms (PACS-Interview) was applied in *n* = 271 children. Odds ratio with the GENMOD procedure (PROCGENMOD) was used to test if the risk for ADHD, TD, and ADHD+TD in siblings was associated with the related index patients' diagnoses. In order to get an estimate for specificity we compared the four groups for general psychopathological symptoms.

**Results:** Co-existing ADHD+TD in index patients increased the risk of both comorbid ADHD+TD and TD in the siblings of these index patients. These effects did not extend to general psychopathology.

**Interpretation:** Co-existence of ADHD+TD may segregate in families. The same holds true for TD (without ADHD). Hence, the segregation of TD (included in both groups) seems to be the determining factor, independent of further behavioral problems. This close relationship between ADHD and TD supports the clinical approach to carefully assess ADHD in any case of TD.

## Introduction

Attention-deficit/hyperactivity disorder (ADHD) with its problems of attention, general motor restlessness, and impulse control is a developmental disorder with a strong impact on the affected individual's life, including academic difficulties, impaired socialization, and strained parent-child relationships (Wu et al., 2012). Both genetic (Banaschewski et al., 2010; Faraone and Mick, 2010) and environmental (Banerjee et al., 2007) factors play a role in the etiology of ADHD. The disorder is accompanied by various psychiatric disorders (Gillberg et al., 2004). Research on familial underpinning of ADHD co-occurring with tic disorders (TD, a neuropsychiatric movement disorder with sudden, short, non-rhythmic, unintentional muscle twitches or vocalizations) is clinically important, since TD are commonly co-existing with ADHD. While about half of children with TD also meet criteria for ADHD (Freeman, 2007), about 20% of children with ADHD are additionally suffering from TD (Kadesjo and Gillberg, 2001; Rothenberger et al., 2007; Schlander et al., 2011).

O'Rourke et al. (2011), while reporting from earlier studies on ADHD+TD, stated “that the increased frequency of ADHD in relatives of TD probands” may be due to the enhanced heritable risk for ADHD+TD. The risk for ADHD alone was not increased among relatives of TD patients. However, these studies were limited by small sample sizes and lacking of families with probands diagnosed with ADHD-only.

Only Stewart et al. (2006) compared the relatives of four different groups with each other (ADHD+TD vs. TD-only vs. ADHD-only vs. healthy controls) and found that “comorbid ADHD+TD diagnoses in relatives were elevated in all case groups” and they concluded that “there is an increased risk of comorbid ADHD and TD in affected families.” Although this study suggests the existence of familiality of ADHD+TD, the small data base with limited sub-sample sizes demands further evaluation concerning (a) whether the pattern of ADHD+TD co-existence really runs in families and (not tested in former studies) (b) how disorder specific this might be.

Since ADHD + TD is highly important in daily clinical practice (e.g., for evidence based psychoeducation, early prevention and treatment) it needs to be disentangled further. Therefore, it seems to be worthwhile elucidating the familiality of ADHD+TD. While taking an ADHD perspective, this study will extend the small empirical data base in order to better answer question (a) and add new knowledge to (b).

Hence, we are running this study by analyzing for the first time a large sample of ADHD affected children and their (partly also affected) siblings, who took part in the IMAGE-study (Muller et al., 2011a,b). Based on previous findings (see above) we expected as a directed hypothesis (a) higher frequency of ADHD+TD in the similarly stratified siblings group of index patients with ADHD+TD vs. the siblings group with ADHD–TD, supporting the assumption that ADHD+TD may run in families and (b) a higher level of broad band psychopathology symptoms in siblings of index children with ADHD + TD compared to siblings of index children with ADHD–TD. The latter could reflect an estimate of disorder related specificity of our segregational findings (non-directed hypothesis).

## Methods and materials

### Sample

Between 2003 and 2006 families with at least one child with the combined subtype of ADHD (=index patient) and their full siblings (regardless of their possible ADHD-status) were recruited as part of the IMAGE (International Multi-center ADHD Genetics) study. IMAGE is a collaborative study of twelve specialist centers in seven European Countries and Israel. It aims to identify genes that increase the risk of ADHD using linkage and association strategies. As inclusion criteria were used an age of 5–17 years, Caucasian descent, IQ ≥ 70, no diagnosis of autism, epilepsy, general learning difficulties, brain disorders and known genetic disorders. Parent and teacher questionnaires (see below) were applied to screen participants. In screening cases positive for a diagnosis of ADHD, the PACS interview (see below) was conducted. PACS interviewers (child psychiatrists, psychologists) were trained at the London Institute of Psychiatry under supervision of Prof. E. Taylor. Inter-rater agreement for PACS was 0.88 (range 0.71–1.00).

A detailed description of the IMAGE sample (including recruitment and inclusion criteria as well as ethical approval) has already been given in previous reports (Muller et al., 2011a,b). The sample of the present analyses included 2815 individuals consisting of 1209 index patients suffering from ADHD and 1606 of their siblings. The higher number of siblings is due to the fact that some patients had more than one sibling who took part in the study.

The **index patients** (in total *N* = 1209) were allocated to the group ADHD+TD (*N* = 262) if they fulfilled the criteria for both ADHD and TD; if TD-criteria were not fulfilled, they were classified as group ADHD-TD (*N* = 947).

**Figure 1:**
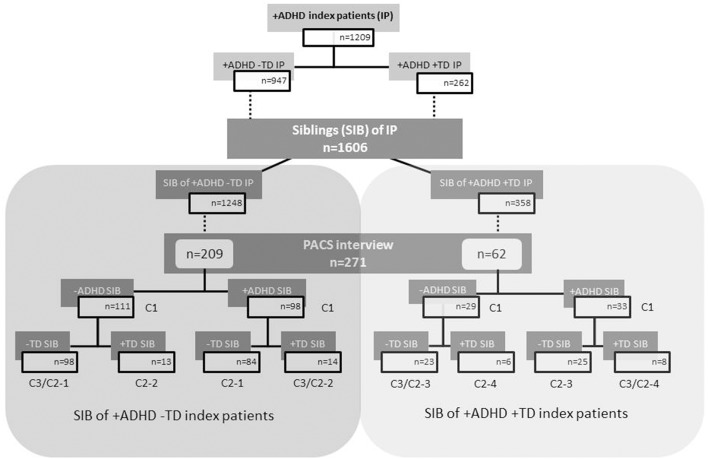
**Flow chart of index patients and their siblings according to ADHD and TD grouping**. C1, C2, and C3 are the numbers of the cells related to the crosstabs 1–3 of Table 1. ADHD, attention-deficit/hyperactivity disorder; TD, tic disorder; IP, index patients; SIB, siblings; +ADHD, with ADHD; −ADHD, without ADHD; +TD, with tic disorder; −TD, without tic disorder.

The **siblings of index patients** (in total *N* = 1606) were differentiated in those of index patients with ADHD–TD (*N* = 1248) and siblings of index patients with ADHD+TD (*N* = 358) regardless of their own diagnostic status. Further the 1606 siblings of index patients with ADHD (i.e., ADHD+TD and ADHD–TD; see above) were screened themselves for ADHD with Conners' Rating Scales and SDQ. In case of scores indicative for a diagnosis of ADHD a PACS interview (see Assessment below) has been conducted and led to a diagnosis of ADHD in 131 siblings. Because the information about a TD diagnosis was taken from the PACS interview and this has been conducted only in those siblings for whom ADHD was assumed by screening, also the information about the occurrence of TD yes/no was available only for 271 siblings (i.e., not all TD siblings without ADHD might have been detected). Among those siblings 41 were identified as being affected with TD (see ‘+TD SIB’ in flow chart Figure 1).

### Assessment

The clinical assessment of dimensional psychopathology was performed with the long versions of Conners' Rating Scales (CRS) for parents and teachers and the Strengths and Difficulties Questionnaires (SDQ) for parents and teachers. Scores were indicative for a diagnosis of ADHD if T-scores were ≥63 on the Conners' DSM-IV ADHD Total score and if the score on SDQ Hyperactivity scale exceeded the 90th percentile. In these cases the valid, standardized semi-structured interview PACS (Parental Account of Childhood Symptoms; Taylor et al., 1986) was executed for the assessment of diagnostic categories. During the PACS parents were asked for detailed descriptions of what their children have done in specified situations over the previous week. Based on these reports, trained investigators verified the ADHD diagnosis according to DSM-IV criteria.

The PACS-interview also allowed the examination of the existence of a TD. For this purpose the parents were asked if their child has ever shown some kind of tic behavior like “sudden, repetitive, stereotyped motor movements, or vocalizations.” In order to be rated in this section, they must, for a period of at least 4 weeks have been occurring many times a day or nearly every day. In case the parents affirmed this question, they were requested to specify what kind of tics they had observed (simple/complex, motor/vocal tics, or both), since when these tics occurred and if the tics have been present in the absence of stimulant medication. According to PACS each DSM-criterion was coded as a dichotomous variable (0 for absent, 1, for present), hence an omnibus diagnosis of TD was given if the child fulfilled the TD criteria. Since TD should be seen along a continuum and to keep large enough TD-subgroups we did not specify for single DSM TD-categories.

### Measurements

To assess the psychopathological profile of the children (index patients and their related siblings) parents and teachers had to complete the long versions of Conners' Rating Scales – Revised (CPRS-R-L and CTRS-R-L, respectively; Conners et al., 1998a,b) and the Strengths and Difficulties Questionnaire (SDQ-P and SDQ-T respectively; Woerner et al., 2004). The CPRS-R-L is a well-established instrument to assess childhood behavior problems. We focused on five of its seven main scales (oppositional, social problems, anxious-shy, psychosomatic, perfectionism) as well as on its DSM-IV scales (DSM-IV inattention, DSM-IV hyperactivity-impulsivity, and DSM-IV total score), to examine the symptomatology of the children. The SDQ is a brief behavioral screening questionnaire with five primary scales (hyperactivity, conduct problems, peer problems, emotional problems, and prosocial problems) which were all considered. All subjects had an estimated full-scale IQ above 80 (Sattler, 1992; Tewes et al., 1999).

### Data analyses

All data were statistically analyzed with SAS (Statistical Analysis System).

To analyze the **familial occurrence** for each of the three conditions under investigation (ADHD, TD, ADHD+TD) we calculated the relative risk of sibling's diagnoses in relation to both index patient-groups (ADHD-TD and ADHD+TD). The odds ratio served as a measure for the degree to which the relative risks of sibling's diagnosis differed between both patient-groups. The method used was PROC GENMOD.

To analyze the level of **psychopathological symptoms** a mean score was calculated for each Conners' and SDQ scale named above. Because of some missing data from questionnaires and PACS interview the number of cases varied between the different psychopathological variables.

An analysis of variances was conducted, including two factors: “proband-status” (index patients vs. siblings) and “disorder” (ADHD–TD vs. ADHD+TD). Index patients and siblings derived from the same families, so their data were partly dependent of each other. But they did not form a sample of matched pairs with one sibling being assigned to each index patient, because number of participating siblings per family varied. Therefore, we employed PROC GENMOD for generalized linear models—a SAS procedure able to handle partly dependent data. Additionally, follow-up comparisons analyzed patient-groups and sibling-groups separately. The comparison of both index patients groups was accomplished with *t*-tests for descriptive purposes. For the case of inhomogeneous variances the degrees of freedom were approximated by the Welch-Satterthwaite equation. For comparison of the sibling-groups we used PROC GENMOD.

## Results

### Group characteristics

Patients having ADHD without TD (ADHD–TD: mean age 10.7 ± 2.8 years) did not differ in age from patients with ADHD+TD (mean age 11.1 ± 2.7 years, *p* = 0.06). Additionally, there were no differences in IQ between the two groups (ADHD–TD: mean IQ = 99.8 ± 16.2 vs. ADHD+TD: mean IQ = 100.0 ± 15.6, *p* = 0.88). However, in the group of children with ADHD-TD the proportion of girls (14.5%) exceeded their proportion in the ADHD+TD-group (6.8%, *p* < 0.01).

Siblings of index patients with ADHD-TD had a mean age of 10.8 ± 3.4 years and a mean IQ of 101.7 ± 13.3. This did not differ from the mean age and IQ of the siblings whose related index patient suffered from ADHD+TD (mean age 10.7 ± 3.4 years, *p* = 0.42, and mean IQ 102.9 ± 13.3, *p* = 0.41). Also, there was no significant difference in gender between both groups (siblings of ADHD index patients 49.9% female vs. siblings of ADHD+TD index patients 52.0% female, *p* = 0.53).

### Frequency of disorder

This analysis included different subsamples (see Figure 1) of siblings, because information about the occurrence of a TD diagnosis was not available for all. Therefore, each subsample of siblings of ADHD–TD patients and each corresponding subsample of siblings of ADHD+TD patients were compared in terms of age, gender and IQ. No significant differences between the quasi-experimental-conditions were found in any of siblings' subsamples.

For the first analysis of familial transmission the relative risk for ADHD did not differ between siblings of index patients with ADHD+TD and siblings of index patients suffering from ADHD without TD (*p* = 0.38, see Table 1, crosstab 1).

**Table 1:** **Frequency of siblings' ADHD and/or TD diagnoses independently of further diagnoses based on Parental Account of Children's Symptoms (PACS) interview**.

**Patients**	**Siblings**		**Relative risk**	**Odds ratio (OR)(*p*-value)**	**(OR)-Confidence interval**
**CROSSTAB 1**					
	no ADHD^a^	ADHD^a^			
ADHD	111	98	0.88	1.29 (0.38)ns	
ADHD+TD	29	33	1.14		0.73–2.27
**CROSSTAB 2**					
	no TD^b^	TD^b^			
ADHD	182	27	0.15	1.97+(0.07)	
ADHD+TD	48	14	0.29		0.96–4.04
**CROSSTAB 3**					
	no ADHD/no TD	ADHD+TD			
ADHD	98	14	0.14	2.43^*^(0.04)	
ADHD+TD	23	8	0.35		0.91–6.49

*Siblings were screened for a previous ADHD diagnosis, clinical suspicion of ADHD, an average T-score of the DSM-IV total symptom score (N-scale) greater than 63 on the Conners scales or scores >90th percentile on the SDQ-hyperactivity scale. Only in verified cases the semi-structured PACS interview was performed leading to diagnosis based on operational DSM-IV criteria for ADHD and/or TD*.

a *Independently of TD status of the siblings*.

b *Independently of ADHD status of the siblings*.

*Data in cells: absolute frequencies*,

* *p ≤ 0.05, ^+^p ≤ 0.10, ns: non-significant*.

*Crosstab 1 and 2: two-tailed; non-directed hypothesis*.

*Crosstab 3: one-tailed, directed hypothesis*.

*The numbers of the cells related to the crosstabs can also be found in Figure 1 as C1, C2, and C3*.

For the second analysis, the relative risk for TD tended to differ between siblings of index patients with ADHD+TD and siblings of index patients suffering from ADHD without TD (*p* = 0.07, see Table 1, crosstab 2).

For the third analysis, siblings of index patients with ADHD+TD had a statistically significant 2.43-fold higher risk for ADHD+TD than siblings of index patients with ADHD without TD (*p* = 0.04, see Table 1, crosstab 3).

### Level of psychopathology

The two-factorial analyses of variance revealed a significant main effect for “proband-status” with regard to all of the psychopathological symptoms. In contrast, no main effect for “disorder” was found except for prosocial behavior rated by parents. The interaction effect was significant on four scales of the CPRS-R-L (oppositional: *p* < 0.01, social problems: *p* < 0.01, anxious-shy: *p* < 0.01, perfectionism: *p* = 0.01) and on three scales of the SDQ-P (conduct problems: *p* = 0.01, peer problems: *p* = 0.01, emotional problems: *p* = 0.01). In teacher ratings the interaction only reached significance on the CTRS-R-L DSM-IV total score (*p* = 0.05; see Table 2).

**Table 2:** **Psychopathological profile**.

	**ADHD patients**		**ADHD+TD patients**			**ADHD siblings**		**ADHD+TD siblings**			**Two-factorial analysis**		
											**Disorder**	**Proband-status**	**Inter-action**
	***M***	***SD***	***M***	***SD***	***t***	***M***	***SD***	***M***	***SD***	***Z***	***Z***	***Z***	***Z***
**CRS-PARENTS**													
Oppositional behavior	70.5	12.4	73.3	11.8	−3.37^**^	55.6	13.4	54.6	12.0	0.94	1.12	20.35^**^	−3.49^**^
DSM-IV inattention	70.9	9.0	71.1	8.1	−1.51	54.9	12.5	55.5	12.6	−0.88	−0.87	19.03^**^	−0.18
DSM-IV hyperactiv-impulsiv	80.6	10.3	82.0	8.5	2.33^*^	56.6	14.7	56.2	14.1	0.11	0.27	28.89^**^	−1.58
DSM-IV total	77.6	9.0	78.9	8.1	−2.20^*^	56.4	12.6	56.4	13.0	−0.63	−0.54	20.29^**^	−0.81
Anxious/shy behavior	58.7	13.8	63.1	14.4	−4.42^**^	53.1	12.1	53.0	12.1	−0.50	−0.21	9.69^**^	−3.65^**^
Psychosomatic symptoms	59.7	15.2	61.6	16.4	−1.68^+^	53.8	13.3	54.2	14.3	−0.44	−0.49	6.18^**^	−1.08
Perfectionism	55.7	11.9	58.1	13.0	−2.67^**^	49.8	9.3	49.4	9.2	0.87	0.93	10.17^**^	−3.10^**^
Social problems	67.0	15.0	71.0	14.5	−3.92^**^	53.4	11.8	53.4	11.6	0.00	0.03	16.39^**^	−3.29^**^
**SDQ-PARENTS**													
Hyperactivity	8.4	1.7	8.7	1.5	−2.43^*^	3.4	3.1	3.4	3.0	−0.20	−0.09	27.14^**^	−1.10
Conduct problems	4.6	2.4	5.1	2.4	−2.94^**^	2.9	2.1	2.9	1.7	0.84	1.00	18.91^**^	−3.19^**^
Emotional problems	3.7	2.5	4.3	2.6	3.22^**^	2.4	2.3	2.3	2.4	0.23	0.34	10.84^**^	−3.07^**^
Peer problems	3.9	2.6	4.5	2.7	−3.37^**^	1.7	1.9	1.7	2.0	−0.42	−0.29	14.69^**^	−2.71^**^
Prosocial behavior	6.9	2.3	6.3	2.3	3.08^**^	8.1	2.0	7.9	2.1	1.81^*^	1.77^*^	−8.57^**^	1.23
**CRS-TEACHER**													
Oppositional behavior	66.8	14.4	64.9	13.8	1.89^+^	55.3	12.9	54.8	12.8	0.56	0.61	9.94^**^	1.13
DSM-IV inattention	63.2	10.5	61.9	10.6	1.73^+^	54.8	11.4	55.3	11.8	−0.64	−0.63	7.46^**^	1.78^+^
DSM-IV hyperactiv-impulsiv	71.7	11.9	69.6	11.0	2.64^**^	56.6	13.0	55.9	13.0	0.67	0.85	14.00^**^	1.19
DSM-IV total	70.8	10.6	68.7	10.3	2.80^**^	55.7	13.8	56.1	13.8	−0.09	−0.00	13.28^**^	1.93^*^
Anxious/shy behavior	65.0	12.8	63.7	11.9	1.55	59.2	12.6	58.4	11.7	0.90	1.00	5.81^**^	0.51
Perfectionism	56.9	11.6	55.8	11.4	1.33	52.4	9.4	52.2	9.2	0.52	0.66	4.46^**^	0.73
Social problems	60.0	13.7	61.1	13.4	−0.51	52.9	11.5	52.6	10.8	0.26	0.51	8.41^**^	−0.76
**SDQ-TEACHER**													
Hyperactivity	7.8	2.1	7.7	2.2	0.88	3.8	3.1	3.8	3.1	0.43	0.51	19.10^**^	0.15
Conduct problems	3.2	2.3	3.0	2.5	1.13	1.4	1.9	1.4	1.8	0.73	0.82	9.86^**^	0.51
Emotional problems	3.0	2.4	2.7	2.3	1.94^*^	2.0	2.2	1.8	2.1	1.42	1.48	5.17^**^	0.61
Peer problems	3.1	2.5	3.3	2.7	−0.97	1.7	2.0	1.5	1.8	1.39	1.57	9.80^**^	−1.79^+^
Prosocial behavior	5.7	2.7	5.2	2.6	2.84^**^	7.3	2.4	7.1	2.5	0.88	0.87	9.54^**^	1.71^+^

*CRS, Conners' Rating Scales – Revised (Conners et al., 1998a,b)*.

*SDQ, Strengths and Difficulties Questionnaire (Woerner et al., 2004)*.

*M, mean; SD, standard deviation; t, test statistic (t-test); Z, test statistic (PROC GENMOD)*.

** *p ≤ 0.01*,

* *p ≤ 0.05*,

+ *p ≤ 0.10*.

The pairwise comparisons showed significant differences between index-patients with ADHD-TD and those with ADHD+TD on most of the psychopathological measures. Parents rated ADHD+TD children as more impaired than children suffering from ADHD-TD. In contrast to the parents' assessment, teachers rated patients with ADHD-TD as showing significantly more severe symptoms of psychopathology than patients suffering from ADHD+TD. For parent as well as teacher ratings effect-sizes were small ranging from 0.05 to 0.32. There were no significant differences between the still normal-range values of their siblings (see Table 2).

## Discussion

The main aim of our study was to investigate the familiality of comorbid ADHD+TD. Hence, we examined the frequency of occurrence of three disorders, namely ADHD, TD, and ADHD+TD in full siblings of index patients with ADHD-TD respective ADHD+TD. Both groups were investigated in a large European sample of ADHD-affected families (Muller et al., 2011a,b). In order to consider the issue of disorder related specificity of probable familiality effects, we also compared general psychopathology between groups.

### Frequency of disorder

We found that the familial risk to develop ADHD was not different in both siblings groups (i.e., siblings of ADHD-index-patients with vs. without TD). In other words, if TD is added to ADHD (in the ADHD+ TD groups) it does not seem to increase the risk to develop ADHD. It follows, that in this case ADHD is the leading vulnerability marker.

In contrast, and this is *our main finding*, there was a significantly higher risk to present with ADHD+TD (and a tendency for TD without ADHD) among siblings of patients with ADHD+TD. This suggests that the familiality of ADHD+TD may exist and is probably driven by the factor TD, which is part of both groups, namely ADHD+TD and TD without ADHD.

To our knowledge there is only one study which examined the occurrence of ADHD+TD in relatives of ADHD+TD-patients (Stewart et al., 2006). It included 239 probands (mean age 13.8) and 692 first-degree relatives in total. The number of cases in each proband-group varied between 41 and 75 and between 114 and 219 in the relative-groups. Stewart et al. (2006) reported that ADHD+TD was increased not only in relatives of the ADHD+TD group but also in relatives of the TD-only and ADHD-only case groups supporting the assumption of a cross-disorder vulnerability and familiality of ADHD+TD. However, in Stewart et al. (2006) (see data adaptation in Table 1 of O'Rourke et al., 2011) the first degree relatives of the ADHD+TD proband groups vs. the ADHD-only group show higher frequencies for TD as well as ADHD+TD. Including our data, it seems probable that TD might be the essential factor for the familiality of both TD and ADHD+TD; i.e., so far there is no clear evidence for a strong common vulnerability of ADHD and TD, respectively. Hence, the “true comorbidity” explanation of ADHD+TD is suggested, which is supported by psychopathological, neuropsychological, and neurophysiological findings pointing merely to an association than to a clinical entity of ADHD+TD (Banaschewski et al., 2007; Rothenberger and Roessner, 2013).

### Level of psychopathology

Parallel to the analysis of the index patients' categorical psychopathology by diagnoses, we assessed the dimensional psychopathological profile in the patients' siblings in order to get a rough estimate of specificity. We did not find any differences comparing the values of siblings of ADHD–TD patients vs. siblings of ADHD+TD patients which all were within the normal range. Thus, the assumption of higher general psychopathological vulnerability in siblings of index patients with ADHD+TD (as a first hint of familiality) was not supported on the basis of dimensional psychopathology.

In affected children teachers' ratings were found somewhat opposite to parents' ratings. While parents rated affected index children with ADHD+TD (compared to ADHD–TD) higher, teacher tended to see more problems in the ADHD–TD than in the ADHD+TD group. Since the effect-sizes of both kinds of differences were small (0.05–0.32) this may underline that TD usually adds little to psychic problems when it co-exists with ADHD (Rothenberger and Roessner, 2013).

Although there are several reports about low agreement between parents' vs. teachers' ratings of ADHD core-symptoms, this issue is still not resolved (Gadow et al., 2008), but one important factor may be, that parents and teachers observe genuinely different settings and thus different kinds and levels of behavior. While parents seem to have a broader, more sensitive view, teachers observational window is smaller and merely specific. In our case it is clinically a well-known fact that children with TD (including those with ADHD+TD) are very often able to control their tics at school and “let them out” when they are at home. Therefore, in ADHD+TD specifically hyper-motor behavior might be hidden at school and quite obvious at home. Thus, when comparing teacher and parent ratings of ADHD–TD vs. ADHD+TD patients, environmental/informant influences might explain the difference with somewhat higher scores in parent ratings.

## Limitations

Some limitations of the study need to be mentioned.

There may exist some diagnostic uncertainty for TD, since (a) TD was diagnosed by parent interview only, (b) the TD group was not differentiated for the known TD categories because there was no full formal assessment and resulted sample size would have been too small for statistical testing. However, percentage of TD (13 vs. 22%) in the respective for ADHD screen positive sib-groups corresponds with the usual frequency of TD in ADHD (Kadesjo and Gillberg, 2001; Rothenberger et al., 2007; Schlander et al., 2011). (c) Because of the two step diagnostic procedure, those siblings with a negative screen for ADHD were not assessed with PACS. Hence, not all TD siblings without ADHD were detected and included. Given that the general population prevalence of about 4% for TD also holds for the ADHD screen negative siblings group we might have missed about fifty children with “TD-without ADHD.”

The gender ratio differed between both index patients groups. Among the patients suffering from ADHD+TD there was a higher percentage of boys compared with the ADHD–TD group. Although this seems to be a natural fact of ADHD+TD (Rothenberger et al., 2007; Schlander et al., 2011) this might have somewhat biased the results toward externalizing behaviors, because ratings of psychopathological symptoms are not independent of gender.

As we analyzed index patients and siblings sharing family environment only interpretations referring to familiality but not heritability and/or genetic background are possible.

Further, our study design included only two index patient-groups (ADHD–TD and ADHD+TD). In order to comment on more specific modes of familiality it would be necessary to additionally include a TD-only and a healthy control-group.

## Conclusions

Our results suggest that ADHD+TD may run in families and that the vulnerability for this might be related to TD. The TD-specificity of our finding is supported by the fact, that TD-only ran also in families and that general psychopathology did not show familiality effects. It remains to be clarified if this familial TD-effect holds in a TD-only group and if it may be influenced by obsessive-compulsive symptoms (which often exist together with ADHD as well as TD), because there is some evidence that association between TD and ADHD “may be due to a genetic association between OCD and ADHD and in part to shared environmental factors” (O'Rourke et al., 2011). Unfortunately, our data set did not allow to test this hypothesis. Whether our significant interaction effect between “proband status” and “disorder” for perfectionism (CPRS-R-L) reflects a signal in this direction remains to be left open. Also, our findings do not allow to firmly conclude if ADHD+TD should best be seen as an additive combination of two separate nosologies or if it should be considered a distinct subtype within a heterogeneous disorder, but recent research at different levels of investigation suggests merely the additive variant (Rothenberger et al., 2007; Rothenberger and Roessner, 2013). Finally, the issue that familiality of ADHD+TD may be driven by TD (independent of further behavioral problems) demands for a careful assessment of ADHD in any case of TD.

## Author contributions

AR, VR, and TB designed and wrote the article; they contributed equally and share main authorship. All authors are principal investigators of the IMAGE study and reviewed and approved the article.

### Conflict of interest statement

The authors declare that the research was conducted in the absence of any commercial or financial relationships that could be construed as a potential conflict of interest.
